# Balancing Risks and Opportunities: Data-Empowered-Health Ecosystems

**DOI:** 10.2196/57237

**Published:** 2025-03-25

**Authors:** Lan Li, Emma Back, Suna Lee, Rebecca Shipley, Néo Mapitse, Stefan Elbe, Melanie Smallman, James Wilson, Ifat Yasin, Geraint Rees, Ben Gordon, Virginia Murray, Stephen L Roberts, Anna Cupani, Patty Kostkova

**Affiliations:** 1 University College London London United Kingdom; 2 World Organisation for Animal Health Paris France; 3 University of Sussex Brighton United Kingdom; 4 Our Future Health London United Kingdom; 5 UK Health Security Agency London United Kingdom

**Keywords:** health policy, data sharing, digital healthcare, healthcare system, ecosystems, technologies, decision-making, data privacy, data protection, social media, application programming interfaces

## Abstract

This viewpoint paper addresses the ongoing challenges and opportunities within the data-for-health ecosystem, drawing insights from a multistakeholder workshop. Despite notable progress in the digitization of health care systems, data sharing and interoperability remain limited, so the full potential of health care data is not realized. There is a critical need for data ecosystems that can enable the timely, safe, efficient, and sustainable collection and sharing of health care data. However, efforts to meet this need face risks related to privacy, data protection, security, democratic governance, and exclusion. Key challenges include poor interoperability, inconsistent approaches to data governance, and concerns about the commodification of data. While emerging platforms such as social media play a growing role in gathering and sharing health information, their integration into formal data systems remains limited. A robust and secure data-for-health ecosystem requires stronger frameworks for data governance, interoperability, and citizen engagement to build public trust. This paper argues that reframing health care data as a common good, improving the transparency of data acquisition and processing, and promoting the use of application programming interfaces (APIs) for real-time data access are essential to overcoming these challenges. In addition, it highlights the need for international norms and standards guided by multisector leadership, given the multinational nature of data sharing. Ultimately, this paper emphasizes the need to balance risks and opportunities to create a socially acceptable, secure, and effective data-sharing ecosystem in health care.

## Introduction

Data are essential to the delivery of effective health care and the improvement of health outcomes. However, the power of data relevant to health has not been fully realized. There is a need to nurture and develop data-for-health ecosystems to collect and share valuable data through transformative and responsible technological systems in timely, safe, efficient, and sustainable ways that maximize data utility for the public good.

A “data ecosystem” refers to a network of entities engaged in generating, sharing, and using data resources through various technologies [1]. In the context of a data-for-health ecosystem, this network is diverse and decentralized, comprising public and private institutions, as well as individuals acting in various roles such as citizens, patients, and consumers. Data are actively and passively generated across all levels of this ecosystem, intentionally recorded for health purposes (eg, National Health Service [NHS] patient records) or initially generated for different reasons but repurposed for health care improvement (eg, geospatial air quality data shared to assist citizens with respiratory issues). Data management within this ecosystem varies across a spectrum: from tightly controlled data, such as patient records protected by strict privacy regulations and access protocols to ensure confidentiality, to fully open data, such as anonymized census or survey data that are freely accessible to the public for research and policy-making purposes. This range encompasses well-structured databases and unstructured streams from sources such as social media and Internet of Things devices, with knock-on effects for how data can be used.

Electronic devices generate digital traces that contribute valuable insights into personal health, extending beyond traditional patient records to include data held by IT and mobile corporations, and even loyalty card accounts [2]. The wide spectrum and variety of spatial-temporal geo-located big data streams that are collected include web-based search keywords, internet and store-based purchases, sensors and Internet of Things (IoT) data, health and fitness app data, and mobile phone location data [3,4].

The rapid evolution of this ecosystem poses risks related to privacy, data protection and security, transparency, access, and exclusion, as highlighted in the 2014 United Nations report “A World That Counts” [5]. The COVID-19 pandemic heightened concerns, due to the proliferation of systems capturing personal information for public health benefit, but often at the expense of robust debate about privacy, data ownership, and technology regulation.

Despite the potential for leveraging digital traces in health care, limitations persist, revealing gaps and missed opportunities [6]. The lack of interoperability between platforms and databases hinders data sharing across sectors, disciplines, countries, and international agencies. While such data holds substantial value for individuals, clinicians, health system planners, policy makers, researchers, and innovators, restrictions on visibility, access, and sharing impede its full potential. For instance, integrating data from health and fitness apps with electronic patient records could enhance clinical understanding, but business interests often deter app companies from developing interfaces for seamless integration.

Beyond interoperability, cross-border data sharing presents another critical hurdle, particularly in addressing global health challenges. Despite its growing importance, legal, regulatory, and technical barriers continue to limit progress. The lack of harmonized data protection standards across countries complicates the sharing of health data, particularly when navigating diverse privacy regulations like the General Data Protection Regulation (GDPR) in the European Union (EU) and less stringent frameworks in other regions. Countries often have conflicting data protection laws, making it difficult to ensure compliance while enabling international data flows. In addition, concerns over data sovereignty, where countries insist on controlling their citizens’ data within national borders, further limit cross-border collaboration [7,8]. In public health emergencies, such restrictions can delay data access, hindering rapid responses. For example, during the COVID-19 pandemic, differing regulations and limited cross-border collaboration created obstacles for real-time sharing of epidemiological data, impeding global tracking efforts [9].

In 2016, the “Findable, Accessible, Interoperable, Reusable” principle was introduced as a guide for improving data reusability, focusing on both machine and human accessibility by stakeholders from academia, industry, funding agencies, and scholarly publishers [10]. Nevertheless, government agencies encounter significant challenges in leveraging population-level data for decision-making due to issues like delayed and inconsistent official data collection and aggregation, and the cost of accessing commercial datasets. Such difficulties were exacerbated during the COVID-19 pandemic, particularly for agencies in low- and middle-income countries with limited funding and infrastructure [11]. Increasingly, nonofficial data is relied upon to provide timely information. This may be generated by citizens or communities as an evidence base for change or to enhance social accountability, but it is often collected by private providers, with individual consumers contributing their data either actively or passively. Increasingly, multinational technology companies engaging in “massive and passive” data collection are dominating data-for-health ecosystems. This trend was accelerated during the COVID-19 pandemic [12] when social media platforms and search tools such as Twitter, Facebook, Google, and Bing played a pivotal role in providing timely information for researchers to monitor public sentiment and assess intervention impacts [13]. While the technology industry certainly benefits from leveraging personal data for web-based marketing, digital epidemiologists demonstrate how these new data streams can also enhance understanding of citizen behavior, support decision-making, and enable reporting of disease symptoms.

The aim of this viewpoint paper is to explore the key challenges and opportunities within these evolving ecosystems, synthesizing insights from the “Data Enabled Society for Health: Challenges and Opportunities” workshop held in London by University College London. This workshop brought together health care providers, policymakers, technologists, and researchers, to consider how to create a secure and effective data-for-health ecosystem that facilitates the gathering and sharing of valuable data for both individual and population-level health benefits, while effectively managing the associated risks. We suggest that to optimize the use of health data, governments and institutions should work on three overlapping areas: data governance, interoperability, and participation and citizen engagement..

## Navigating the Dual Realities of Data Governance: Public Sector Caution and Industry Data Challenges

Ongoing issues with data governance, data access, and privacy highlighted during the workshop extend beyond regulatory and technical concerns to profound ethical dilemmas. These include the challenge of obtaining meaningful informed consent in complex data ecosystems, the risk of data commodification that reduces individuals to mere data points, and the ethical responsibility of both public and private entities to prioritize the rights of individuals over profit or efficiency. These concerns also intersect with issues of data equity, where marginalized groups may face greater risks of exclusion or exploitation.

Within the public sector, citizens are not the only ones to limit access to their data due to privacy or security concerns. Governmental organizations and health care institutions are also very wary of granting access to their datasets, sometimes even to researchers, fearing either reputational damage due to mishaps or the loss of future financial opportunities if they share their assets.

This protective attitude towards an organization’s data assets reflects the perception of data as a commodity: something that can be “extracted” from individuals or communities and then traded. We think it is important to reframe this narrative and look at personal data in the same way we look at common goods, with regulated access and shared collective benefits [14,15].

Attitudes against sharing medical information can stem from confusing messages and a lack of controls in the past, as well as fear of data being shared with third parties in ways that invade privacy, lead to increased surveillance and authoritarianism, or enable personal data exploitation against the individual’s interests (eg, with an insurance provider). Citizen engagement needs to be specific and honest about the risks and benefits of data sharing, rather than relying on an abstract notion of how data sharing may benefit humankind, to avoid pitfalls such as the failed launch of NHS Care.data initiatives [16] and the abandoned centralized NHS COVID-19 Test and Trace app [17]. At the policy level, the key challenges are increasing the transparency of data governance and developing robust deidentification techniques that preserve the usability of data for research, rather than anonymization. Transparency and open dialogue with citizens are paramount for regaining public trust and setting cornerstones for a balanced agenda. This must be seen as an ongoing process, as trust is never a given.

The above-described scenario of data sharing sits in stark contrast to the vast quantity of personal physiological and health data collected through social media, wearables, tracking devices, MedTech, and geo-located mobile apps. While there are differences in legal frameworks across geographies, for example, in Europe (where GDPR applies, giving more control to citizens over their data), the United States, and China, personal data are generally subject to industry-defined terms and conditions, often with no clear or accessible opt-out clauses allowing use for personalized web-based or mobile marketing. This happens without much awareness or evident concern from citizens and in the absence of robust policy debate. To rebalance the current situation, there is an urgent need for international regulation, oversight, and a restoration of user controls over personal data.

The evolving nature of data-for-health ecosystems therefore raises ethical issues that require deeper consideration. These include mechanisms for ensuring informed consent, particularly in settings where data collection occurs passively or through third-party platforms, often without clear communication with individuals. Traditional models of consent are increasingly inadequate in such ecosystems, leading to a growing call for more dynamic or granular consent mechanisms that allow individuals greater control over specific types of data use. Moreover, the balance between individual privacy and public health is a critical ethical dilemma. While sharing health data has clear societal benefits, especially during public health emergencies like the COVID-19 pandemic, it raises the risk of compromising individual privacy. Finding an ethical equilibrium requires robust deidentification techniques, transparent data governance frameworks, and public engagement in decision-making processes. Finally, we must address the ethical issues of data commodification, where individuals’ health data is treated as a commercial product. This commodification risks not only violating privacy but also entrenching inequalities, as those with fewer resources may have less control over their data and greater exposure to exploitation.

## Ending Data Silos: Radical Interoperability and Real-Time Access

In addition to governance and access to data, the lack of common platforms and standards for data sharing limits collaboration across institutions and countries. This can have potentially devastating consequences in public health emergencies when a rapid and coordinated response is needed.

A notable example of successful collaboration is the Innovative Medicines Initiative ConcePTION consortium (2019), a European partnership involving public and private organizations that collect or access data related to pregnancy, childbirth, and lactation. Innovative Medicines Initiative ConcePTION aims to enhance the availability of health data and transform it into actionable evidence to improve clinical practices and outcomes [18]. Another valuable framework is the Unified Information and Interoperability Governance model, which integrates principles of both information governance and interoperability. The Unified Information and Interoperability Governance framework was designed to improve adaptability, flexibility, and efficiency in health information usage across the entire health care system [19]. Similarly, the United Nations Educational, Scientific and Cultural Organization - Committee on Data for Science and Technology (UNESCO-CODATA) Global Project contributes to the UNESCO Open Science Toolkit by providing comprehensive guidance, checklists, and factsheets specifically aimed at fostering data transparency, accessibility, and collaboration during crises [20]. The project delivers tools for both policy makers and scientists to support data collection, management, and governance. Such frameworks could be instrumental in future health crises, aligning with the goals of creating more interoperable and transparent data ecosystems.

One underused approach to health data access is that of application programming interfaces (APIs), which facilitate real-time, secure data sharing between systems. When integrated with emerging technologies like blockchain and federated learning, APIs can support more secure, privacy-preserving, and decentralized data exchanges. Blockchain can provide the security backbone for APIs, ensuring that data integrity and ownership are maintained, while federated learning can allow institutions to collaborate on data analytics without sharing sensitive raw data. Combined with machine learning algorithms, APIs can further enhance real-time data analysis, enabling rapid public health responses based on predictive insights. This is of particular importance for real-time data streams, such as those generated by social media or IoT sensors. An example of this approach was Twitter allowing free API access to 1% of its database—a bold move that provided real-time data and fueled thousands of research projects in social computing, epidemic intelligence, sentiment analysis, and network and graph computing over a decade [21]. It was also incorporated into the epidemic intelligence platforms used by the World Health Organization (WHO) and its member states, such as MediSys, Epidemic Intelligence from Open Sources (EIOS), and Global Public Health Intelligence Network. This provision ceased under Twitter’s new ownership [22]. Free access to the Twitter API was suspended in February 2023, causing significant disruption to academic research that relied on social media data [23]. Other examples of real-time data gathering and integration platforms include the flights portal Skyscanner and the financial products portal MoneySuperMarket. However, real-time data access for health applications is lacking.

Blockchain offers a groundbreaking solution for managing health data by using distributed, immutable ledgers that enhance transparency, security, and trust. In a data-for-health ecosystem, blockchain technology ensures that health data remains unaltered and that individuals retain control over their personal information through the use of smart contracts. These smart contracts facilitate secure, automated agreements between data owners and users, ensuring that data sharing complies with predefined privacy rules. The decentralized nature of blockchain makes it especially suitable for secure cross-border data sharing among institutions, allowing real-time access while safeguarding data integrity. Moreover, blockchain can support granular consent mechanisms, enabling individuals to specify who can access their data and under what conditions. A notable example is Estonia’s health care system, which has successfully integrated blockchain technology to secure over 1 million patient records, ensuring both data privacy and transparency [24].

Meanwhile, machine learning (ML) is revolutionizing health care by enabling the analysis of large and complex datasets to identify patterns, predict outcomes, and support better decision-making. In data-for-health ecosystems, ML can be used to analyze real-time data streams from various sources, such as social media, wearable devices, and electronic medical records, to predict public health trends like disease outbreaks or shifts in vaccination behavior. ML algorithms enhance real-time epidemiological surveillance, facilitating the early detection of potential health threats through the analysis of social media activity, internet search trends, or mobile health app data. This predictive capability aids in timely public health interventions and resource allocation. For instance, Google’s DeepMind has applied ML to the NHS to improve diagnostic accuracy and predict patient outcomes by analyzing historical health records [25].

Other innovative methods enable ML models to be trained on decentralized data without requiring the sharing of raw data between institutions. Federated learning allows health care institutions to collaborate on model development while maintaining their data locally, thus ensuring data privacy and compliance with regulatory standards like the GDPR. It promotes interoperability without compromising data ownership, making it ideal for cross-institutional collaborations. For example, federated learning has been successfully implemented in medical imaging projects, improving disease detection without the need for direct data sharing across institutions [26]. Similarly, zero-knowledge proofs offer a method for verifying the truth of information without revealing the underlying data. This can significantly enhance data-for-health ecosystems by allowing health institutions to share valuable insights while preserving the privacy of sensitive patient information. For example, health systems could confirm the validity of vaccination status or other health data without exposing any personal identifiers. Cryptographic zero-knowledge proof systems have been explored in secure identity verification services, such as confirming COVID-19 results, while keeping patient identities confidential [27]. In addition, the emerging role of the Metaverse in health care is gaining attention, as its potential applications continue to grow with advancements in Web 3.0 technology [28]. The Metaverse offers an immersive, interoperable ecosystem that could redefine the traditional health care system through avatar-based meetings, simulations, and social interactions between patients, providers, and organizations. However, technological innovation, regulatory oversight, and sound governance are needed to address current challenges in its development.

## Winning Citizen Trust and Public Engagement

Limited access to technology in certain sectors of society and parts of the world can be attributed to resource and infrastructure constraints. Collaborative efforts across governments and communities are essential to address these challenges and prevent exclusion based on factors such as age or socioeconomic status [29].

Recently, concerns about data use and potential misuse have intensified due to health care provider hacking incidents and a lack of transparent communication regarding consequences and damages [30]. Case studies such as the Care.data program [14] and General Practice Data for Planning and Research [30] underscore the critical role of public trust in the success of health data initiatives. Public skepticism, evidenced by opt-out rates and project pauses, demonstrates the need for responsible data use [31].

Diminishing public trust, exemplified by incidents like the NHS Royal Free Trust data privacy breach, has profound consequences for current and future health data initiatives [30]. To foster meaningful citizen participation, mechanisms such as multistakeholder platforms, cocreation exercises, and public consultations can be used. Engaging the public in deliberative discussions, as demonstrated by the Understanding Patient Data (UPD) initiative, is crucial [32]. Through cocreative workshops [33], UPD facilitates meaningful discussions among diverse stakeholders, ensuring public perspectives and concerns are not only heard but also integrated into decision-making processes [34]. This inclusive approach cultivates trust in responsible data usage and ensures citizen expectations shape data governance practices. Collaborative governance models offer a structure where stakeholders can jointly manage and govern data processes [35]. These models emphasize shared accountability, transparency, and continuous feedback, helping to mitigate power imbalances between different groups. Such models can be adapted to include conflict-resolution strategies to address disagreements or tensions that may arise during the decision-making process. Furthermore, achieving alignment across disparate groups requires consensus-building strategies. Techniques such as participatory decision-making frameworks and deliberative processes can help in forming agreement on key issues such as data ownership, privacy, and access rights [36].

Another notable initiative that frames health data as a common good is the Our Future Health program delivered in collaboration with the NHS. This program aims to collect health information from up to 5 million adults across the United Kingdom on a voluntary basis [37]. By September 2024, more than 1.8 million people had taken part. Researchers from universities, charities, the NHS, and health-related companies can apply for access to Our Future Health resources. It is anticipated that this will enable them to explore and test innovative approaches for predicting, detecting, and treating common diseases such as dementia, cancer, diabetes, heart disease, and stroke [37].

In summary, Textbox 1 outlines the key future directions, recommendations, and research agenda emerging from the workshop discussions.

**Future directions, recommendations, and research agenda.**

**Research priorities**

Several key areas require further investigation to advance the development of robust and secure data-for-health ecosystems:

- Data privacy and security: Future research should focus on developing and refining privacy-preserving technologies that enable secure data sharing without compromising individual privacy. Researchers should also explore the effectiveness of granular consent mechanisms in empowering individuals to control their data.
- Interoperability: Addressing the lack of interoperability between health care systems, both within and across national borders, is vital. Building on successful interoperability and disease coding standards such as SNOMED-CD (Systematized Medical Nomenclature for Medicine–Clinical Terminology) and *ICD-10 (International Statistical Classification of Diseases and Related Health Problems, 10th Revision)* [38,39], research should prioritize the development of standardized data-sharing protocols and application programming interface–based solutions that can facilitate real-time data exchange while ensuring compliance with differing regulatory frameworks.
- Ethical governance models: As data-for-health ecosystems evolve, the development of inclusive governance models that balance the interests of diverse stakeholders will be essential. Researchers should focus on designing collaborative governance structures that integrate citizen engagement and address concerns about data commodification and trust.
- Cross-border data sharing: Given the global nature of health challenges, it is crucial that future research addresses solutions for overcoming the legal and regulatory barriers to cross-border data sharing. This effort should focus on developing and endorsing international agreements, as well as harmonizing standards, to ensure the secure and ethical exchange of health data. Key stakeholders in this process should include organizations such as the World Health Organization, European Commission, and national Ministries of Health. An important step in this direction is building on existing frameworks, like the European Health Information Gateway [40], to facilitate greater collaboration. In addition, fostering partnerships between the World Health Organization, the Association of European Operational Research Societies, and the European Centre for Disease Prevention and Control could further enhance data accessibility, with the creation of a joint data portal and application programming interface for developers to access health data securely and seamlessly.

**Recommendations for policy makers**

Policy makers play a crucial role in shaping the future of data-for-health ecosystems. While this is the hardest challenge to address due to the varying priorities of member states and IT industry commercial pressure We recommend the following actions:

- Regulatory harmonization: policy makers should work toward the harmonization of data protection standards globally, to facilitate cross-border data sharing while safeguarding privacy. Collaborative efforts between international organizations and governments are key to establishing global frameworks for data governance.
- Incentivizing technological innovation: governments should provide incentives for the adoption of privacy-enhancing technologies and interoperable data systems, including the use of federated learning and blockchain for secure data sharing.
- Public trust and engagement: public trust in data systems must be a priority. Policy makers should encourage the development of transparent governance frameworks that involve citizens in decision-making processes and ensure public accountability in the use of health data.

**Guidelines for practitioners and researchers**

Health care practitioners and researchers should focus on the practical implementation of new technologies and frameworks. We recommend:

- Adopting best practices for data sharing: health care institutions should adopt best practices for data sharing that prioritize data security, interoperability, and patient consent, while supporting the reuse of anonymized and pseudonymized data for research, policy, and social good. These practices can be informed by pilot projects and case studies that have successfully integrated innovative technologies such as machine learning and federated learning into health systems.
- Collaborative research initiatives: researchers should pursue collaborative initiatives that bring together stakeholders from academia, industry, and government to cocreate solutions. These collaborations will be critical for testing the feasibility and effectiveness of emerging technologies in real-world settings.
- Focus on equity and inclusion: researchers and practitioners must ensure that the benefits of data-for-health ecosystems are equitably distributed. This includes prioritizing access to technology for underrepresented populations and ensuring that governance models include mechanisms for inclusive participation.

## Conclusion

Perspectives on the risks and opportunities within a data-for-health ecosystem vary based on the vantage points of government agencies, private entities, and individual citizens. Balancing top-down regulation with bottom-up citizen engagement is crucial for enabling data generation, usage, and sharing while safeguarding individuals. Examining successful practices, such as the NHS’s Data Utility framework, and the case studies (Textbox 2) highlighted in this paper can inform the development of adaptable models across countries.

**Case studies in data-for-health ecosystems.**

**Successful interoperability: The COVID-19 vaccination rollout in Israel [41]**

Israel’s national health infrastructure enabled the rapid, seamless integration of vaccination data across health care providers during the COVID-19 pandemic. Interoperability between public health databases and electronic medical records allowed real-time data sharing, which facilitated efficient tracking, resource allocation, and public health decision-making. This success was driven by a well-established national health database and the effective use of application programming interfaces (APIs) that allowed data exchange between different systems. The case highlights how clear governance and technical standards can result in radical interoperability and improve public health outcomes.

- Key factors for success:
  A centralized national database, clear data-sharing protocols, and government-driven API adoption.
- Lessons learned:
  This case aligns with our proposed interoperability suggestions by demonstrating the value of standardized APIs and centralized databases in promoting real-time data sharing.

**Interoperability challenges: Data sharing during the Ebola Epidemic (West Africa, 2014-2016) [42]**

During the 2014-2016 Ebola epidemic in West Africa, the lack of interoperability between different health care systems and organizations severely hindered real-time data sharing and coordination among international health agencies. Various platforms were used to track the epidemic, but without standardized protocols, the response was delayed, costing lives. This case demonstrates the dangers of fragmented data systems during public health emergencies.

- Key factors for failure:
  Lack of standardized data-sharing protocols, limited use of APIs, and a fragmented health infrastructure.
- Lessons learned:
  The case reinforces the importance of adopting standardized interoperability solutions, such as APIs, to enable real-time data sharing across countries and organizations.

**Governance challenges:** **The failure of the National Health Service Care.data program (United Kingdom) [43]**

The NHS Care.data initiative, launched in 2014, intended to pool health data across general practices to improve patient care and medical research. However, the initiative faced severe backlash due to inadequate communication, a lack of clear consent mechanisms, and fears over data commodification. Concerns about patient privacy and the potential for data misuse led to public outcry, and the program was ultimately abandoned in 2016.

- Key factors for failure:
  Poor communication, lack of transparent consent processes, and public distrust.
- Lessons learned:
  This case emphasizes the need for clear, dynamic consent mechanisms and transparent governance practices. It also underlines the importance of trust-building initiatives before embarking on large-scale health data programs.

**Citizen engagement:** **The Understanding Patient Data Initiative (United Kingdom) [44]**

The Understanding Patient Data initiative in the United Kingdom is an example of effective citizen engagement in data governance. By involving the public in cocreation workshops, the initiative has fostered dialogue on how patient data should be used, addressing concerns about privacy and data ownership. Through transparency reports and educational programs, this initiative has built a stronger foundation of trust and enabled more meaningful public participation in decision-making about health data.

- Key factors for success:
  Transparent communication, active public involvement, and education initiatives.
- Lessons learned:
  This case supports our proposed citizen engagement advocacy, particularly the importance of transparency and cocreation in building trust in data governance.

The innovative deployment of transformative technologies in dynamic data-for-health ecosystems is a far-reaching goal. However, the international agenda must also focus on equity, access to technology, and IT literacy, embracing the needs of citizens of all ages and in all regions of the world, to avoid a growing digital divide. Furthermore, international norms and standards are required, given the burgeoning role of multinational companies in this space and the vital importance of transboundary data sharing when addressing public health emergencies of international concern.

Global collaboration plays a pivotal role in advancing standardized data-sharing frameworks, ensuring interoperability, and fostering mutual trust among nations. Establishing robust international partnerships can accelerate the adoption of common standards while respecting local contexts and priorities. Such collaboration will also help to harmonize efforts toward equitable access to technology and shared public health goals. Large institutions have a key role in shaping the direction of this ecosystem and steering policies in specific directions. For example, the rapid and ubiquitous growth of efficient data collection tools—which have become an essential part of our lives—prompted the EU to devise the GDPR framework that became law in 2018. Due to the size of the market it covers and the EU’s geopolitical role, it has effectively set the standard for data protection and management worldwide, building on the WHO and European Centre for Disease Prevention and Control European Health information Gateway [40]. Besides regulations and limitations, governmental bodies can promote and support private endeavors that create digital tools with a privacy-by-design approach. A recent joint statement—the European Health Data Space proposal—from health care stakeholders emphasizes the need for regulated industry access to health data for innovation and societal benefits, recognizing the interconnected nature of the data-for-health ecosystem [45]. Such initiatives, alongside the recommendations in this paper, offer the potential for effective management of this ecosystem. This is crucial to balance diverse interests and ensure that the value of health data benefits society as a whole.

## Abbreviations

API: application programming interface
EIOS: Epidemic Intelligence from Open Sources
EU: European Union
GDPR: General Data Protection Regulation
IoT: Internet of Things
ML: machine learning
NHS: National Health Service
UNESCO-CODATA: United Nations Educational, Scientific and Cultural Organization - Committee on Data for Science and Technology
UPD: Understanding Patient Data
WHO: World Health Organization
